# Exploring Barriers to Patients’ Progression in the Cardiac Rehabilitation Journey From Health Care Providers’ Perspectives: Qualitative Study

**DOI:** 10.2196/66164

**Published:** 2025-02-21

**Authors:** Shri Harini Ramesh, Darwin Jull, Hélène Fournier, Fateme Rajabiyazdi

**Affiliations:** 1 Department of Systems and Computer Engineering Faculty of Engineering and Design Carleton University Ottawa, ON Canada; 2 National Research Council Canada Moncton, NB Canada; 3 Bruyère Research Institute Bruyère Ottawa, ON Canada

**Keywords:** cardiac rehabilitation, health care providers, CR patient journey, qualitative study, barriers, technology

## Abstract

**Background:**

Cardiovascular diseases are one of the leading causes of mortality globally. Cardiac rehabilitation (CR) programs are crucial for patients recovering from cardiac events, as they help reduce the risk of recurrent events and support patient recovery. The patient’s journey in CR spans the stages before, during, and after the program. Patients have to progress through each stage of CR programs successfully to complete the entire CR journey and get the full benefits of CR programs, but numerous barriers within this journey can hinder patient progression.

**Objective:**

This study aims to explore the barriers to progression at all stages of the CR patient journey from the perspectives of health care providers involved in CR care.

**Methods:**

This qualitative study involved semistructured interviews with health care providers involved in CR care from July 2023 to January 2024. A purposive maximal variation sampling method was used to target providers with diverse demographics and specialties. Snowball sampling was used to recruit participants, leveraging the existing networks of participants. Each interview lasted between 30 and 45 minutes. Interviews were recorded, transcribed verbatim, and analyzed using an inductive thematic analysis approach. Data analysis was conducted from August 2023 to February 2024.

**Results:**

Ten health care providers, comprising 7 females and 3 males, were interviewed. Their roles included physician, program director, nurse manager, clinical manager, nurse coordinator, nurse, physiotherapist, and kinesiologist. The analysis identified four overarching themes related to barriers to progression in the CR journey: (1) patients not being referred to CR programs, (2) patients not enrolling in CR programs, (3) patients dropping out of CR programs, and (4) patients’ lack of adherence to lifestyle changes post-CR programs.

**Conclusions:**

In light of the growing interest in technological interventions in CR programs, we proposed 4 potential technological solutions to address the barriers to progression identified in our analysis. These solutions aim to provide a foundation for future research to guide the development of effective technologies and enhance patient progression within the CR journey.

## Introduction

Cardiovascular diseases are one of the leading causes of mortality globally [1]. Cardiac rehabilitation (CR) programs are often recommended for patients who have experienced a cardiac event to reduce the risk of recurrent cardiovascular events and support patient recovery [2]. CR programs are comprehensive and multidisciplinary, encompassing exercise training, risk factor management, health education, and psychosocial support [3,4]. Research has shown that participation in CR programs significantly reduces morbidity and mortality associated with cardiac events [5,6] while also enhancing patients’ health-related quality of life and psychological well-being [7,8].

The patient’s CR journey begins even before enrollment and continues through maintaining lifestyle changes after completing the program [9,10]. It begins with a provider’s referral to the CR program, made before discharge from the hospital following a cardiac event or surgery [11]. The journey then involves enrolling in, participating in, and completing the CR program [12]. After completing the program, the journey continues with integrating and maintaining the lifestyle changes and health habits learned throughout the program [13]. Patients must successfully progress through each stage to complete the entire CR journey in order to get the full benefits of CR programs. However, numerous barriers exist within the CR patient journey that could affect progression.

Previous research has identified various barriers in CR, such as low patient interest, comorbidities, travel complications, limited program availability, scheduling conflicts, and financial constraints [14-18]. However, these studies focused only on specific stages of the CR patient journey, such as referral, enrollment, completion, or postcompletion, but not throughout the entire CR patient journey. Understanding the barriers to progression throughout the entire CR patient journey is essential because each stage of the journey is interconnected, and barriers at one stage can influence subsequent stages [19,20]. Moreover, existing studies predominantly focus on the patient’s perspective. It is equally important to consider the perspectives of the providers who deliver CR care, as they encounter a diverse range of patients with varying needs. Insights from those who deliver care can provide valuable information on improving CR delivery and addressing barriers effectively [21].

The aim of this study was to explore the barriers to progression throughout the CR patient journey from the perspectives of health care providers involved in CR care. We conducted semistructured interviews with 10 providers and identified 4 barriers to progression within the CR patient journey. In light of the growing interest in technological interventions in CR programs [20,22], we proposed 4 potential technological solutions to overcome the barriers to progression identified in our study. The novelty of our work lies in the identification of barriers to progression from the perspectives of health care providers and the proposal of potential solutions to address these challenges. We hope these potential solutions provide the foundation for future research and guide the development of effective technologies to overcome barriers to progression within the CR patient journey.

## Methods

### Ethical Considerations

This study protocol was approved by the ethics committees of Carleton University (clearance number 119779) and the National Research Council Canada (clearance number 2023-108). Ethical review was conducted per institutional guidelines, with no exemptions or waivers applied. Written informed consent was obtained before each interview. Participants were informed of their right to withdraw at any time without consequence. No secondary data were used. All data were anonymized to ensure privacy and confidentiality. No compensation was provided to the participants.

### Overview

This qualitative study is reported in accordance with the COREQ (COnsolidated criteria for REporting Qualitative research) guidelines [23] (Multimedia Appendix 1). We conducted semistructured interviews to explore the barriers to progression in the CR patient journey from the perspective of the health care providers involved in CR care. We chose interviews to ensure that participants could freely share their thoughts and experiences. The semistructured format allowed us to ask follow-up questions in order to gain an in-depth understanding of their perspectives. In addition, the confidential nature of the interviews encouraged open and honest sharing [24]. Eligible participants included health care providers who refer patients to CR, those who provide CR services, and those who manage CR programs. Eligible participants were also required to be proficient in English. Exclusion criteria included individuals not involved in direct patient care or CR, such as administrative staff. To ensure that the perspectives of a diverse group of participants from various specialties in CR care were included, we used purposive sampling [25]. To further expand the participant pool, we used snowball sampling methods [26]. HF, who has extensive connections with health care providers involved in CR care and CR centers, shared the study objectives and recruitment details within her network. Through this process, she received the email addresses of potential participants. Subsequently, SHR contacted these potential participants via email, providing detailed information about the study and inviting them to participate. We contacted 11 potential participants, of whom 10 agreed to participate, while 1 did not respond. No participants dropped out after providing informed consent. No prior relationship was established between the researcher and the participants before the study commenced, and participants knew only that the researcher was conducting the study as part of an academic project focused on CR.

### Data Collection

Data collection took place from July 2023 to January 2024. The interviews were conducted virtually via Microsoft Teams, with each session lasting between 30 and 45 minutes. No repeat interviews were carried out. Each participant was interviewed once during the study. The researcher (SHR), a female PhD student familiar with the subject matter and trained in qualitative research, conducted the interviews, asked questions and follow-up questions as needed, and took notes during the sessions. While conducting the interviews, the researcher maintained a neutral stance, ensuring that her personal views or assumptions did not influence the questions asked or the participants’ responses. The research team collaboratively and iteratively developed an interview script and questions (Multimedia Appendix 2). The questions were open-ended to allow for an in-depth exploration of the providers’ perspectives on the barriers to progression in the CR patient journey, with follow-up questions asked when appropriate. We conducted a pilot test of the interview guide with a health researcher to ensure clarity, relevance, and logical flow of the questions. Feedback from this pilot test was used to refine the questions and the interview guide before data collection commenced.

### Data Analysis

Our analysis was conducted from August 2023 to February 2024 inductively and did not aim to validate any preexisting theories or hypotheses. We followed a thematic analysis approach following the 6 steps outlined by Braun and Clarke [27]. Two analysts, SHR and a male undergraduate student (DJ) with subject knowledge and qualitative research training, were responsible for the analysis. Disagreements in coding and theme assignment were resolved through consensus facilitated by senior female researchers FR and HF, both of whom are familiar with the subject matter and hold PhDs with extensive experience in qualitative research. We adopted a reflexive approach, acknowledging the influence of researchers’ views and experiences [28]. In step 1, both analysts read interview transcripts and took notes. In step 2, they reviewed the first 2 transcripts line by line and identified prominent codes to generate a codebook using NVivo software (version 14.23; Lumivero), which was then applied to subsequent interviews. Codes were iteratively refined every 2 interviews. Step 3 involved systematically grouping codes into candidate themes. In step 4, these themes were compared and refined. In step 5, other team members contributed to further theme refinement. Thematic saturation was assessed using a saturation table that documented the themes found in each interview. Finally, in step 6, we produced a report summarizing our findings. Transcripts were not returned to participants for comment or correction. The analysis was conducted based solely on the interview data collected during the sessions.

### Rigor and Trustworthiness

Rigor and trustworthiness were achieved by ensuring credibility, transferability, dependability, and confirmability throughout the research process [29]. To address credibility, we conducted peer debriefing sessions within our research team, which provided an external check on our interpretation of the data, analysis, and coding decisions. Dependability was achieved through detailed documentation of themes and subthemes, providing transparency, and enabling replicability of the coding process. To support transferability, we provided comprehensive descriptions of our study design, participant selection criteria, and data collection methods, allowing others to evaluate the applicability of our findings to various CR settings. As Lincoln and Guba [30] highlighted, confirmability is achieved when credibility, transferability, and dependability are established. We ensured confirmability by documenting the rationale for theoretical, methodological, and analytical decisions throughout the study.

## Results

### Overview

Ten health care providers, comprising 7 females and 3 males involved in CR care, including a physician, a program director, a clinical manager, a nurse manager, a nurse coordinator, a nurse, 3 physiotherapists, and a kinesiologist, were interviewed (Table 1). The thematic analysis revealed four overarching themes concerning barriers to progression within the CR patient journey, as perceived by these providers: (1) patients not being referred to CR programs, (2) patients not enrolling in CR programs, (3) patients dropping out of CR programs, and (4) patients’ lack of adherence to lifestyle changes post-CR programs. Multimedia Appendix 3 provides illustrative quotes within each of the themes identified. Thematic saturation was reached after analyzing 7 interviews [31] (Multimedia Appendix 4). This was determined using a saturation table, where no new themes emerged after the seventh interview. To ensure robustness, 3 additional interviews were conducted, which confirmed the saturation of themes. The early achievement of saturation reflects the shared perspectives of participants, despite their varied roles within CR care.

**Table 1:** Characteristics of interviewed participants.

Characteristics			Values
Total, n			10
**Sex, n (%)**			
	Female	7 (70)	
	Male	3 (30)	
**Specialty, n (%)**			
	Clinical manager	1 (10)	
	Nurse manager	1 (10)	
	Nurse	1 (10)	
	Physician	1 (10)	
	CR^a^ program director	1 (10)	
	Kinesiologist	1 (10)	
	Physiotherapist	3 (10)	
	Nurse coordinator	1 (10)	
**Practice setting, n (%)**			
	Urban	7 (70)	
	Urban and rural	2 (20)	
	Rural	1 (10)	
Age (year), mean (SD)			47.9 (10.2)
Years of experience, mean (SD)			18.7 (9.5)

^a^CR: cardiac rehabilitation.

### Patients Not Being Referred to CR Programs

One of the barriers to progression within the CR patient journey is patients not being referred to CR programs, as without referrals, patients may not have access to CR programs (Table S1 in Multimedia Appendix 5). Unintentional biases in referrals, such as those related to health conditions, geography, age, and gender, can contribute to patients not being referred to CR programs. As one provider noted, “There is a lot of inherent bias in terms of who is being referred to rehab” [P3]. Providers may hesitate to refer patients with comorbidities or complex health conditions to CR programs due to concerns about their ability to participate. In addition, if the patient is not residing near a CR center, the provider may assume that it is difficult for the patient to travel and, as a result, be hesitant to refer the patient to a CR program. Providers may perceive that older adults might be frail or disconcerted after a cardiac event or surgery, which may result in providers not referring these patients to a CR program. Some providers may be hesitant to refer certain patients to CR programs, considering their gender-specific responsibilities, such as work, family, and parental roles, which could contribute to women being overlooked for referrals.

Furthermore, another reason for not referring is the providers’ heavy workloads. The paperwork involved in referring patients to CR programs can be time-consuming, which may discourage some providers from making these referrals. Although automatic referral systems are available in some CR centers, providers’ endorsements are crucial for motivating patients; as one provider emphasized, “Endorsements from [providers] are key for [motivating patients]” [P1]. However, individually discussing the benefits of CR with every patient is time-consuming and requires much effort. Referring providers may also lack awareness of the various programs available at the CR center. As noted, “[Referring providers] just don’t know all the services that are available” [P8]. Consequently, providers may hesitate to refer patients due to uncertainty about the programs available. In addition, providers mentioned that it is important to carefully select a CR program that aligns with the patient’s needs, as some programs with high-impact exercises might be appropriate for immediate needs, whereas low-impact exercise-based programs could offer better long-term benefits. However, identifying the best CR program for each patient during referral can be challenging for providers without access to a wholesome list of CR programs.

### Patients Not Enrolling in CR Programs

Another barrier to progression in the CR patient journey is patients not enrolling in CR programs after referral. After patients are referred, they must enroll and start the program to benefit from it. Providers mentioned various factors contributing to this nonenrollment (Table S2 in Multimedia Appendix 5). Providers often observed that a substantial number of patients lack awareness and knowledge about CR, with many patients being unaware of its importance and benefits. Providers also noted that patients sometimes hold misconceptions about CR, believing that they can recover by themselves at home or that surgical treatment alone is sufficient without rehabilitation. Providers think that inconvenient waiting periods also contribute to patients not enrolling in CR programs. For some patients, the waiting period may be perceived as too short after surgery, leading them to feel unready to start the program. Conversely, for other patients, the waiting period between referral and the program’s start may seem excessively long. In addition, CR programs are sometimes offered on fixed schedules, where groups of patients begin the program simultaneously. If patients miss the upcoming enrollment window, they will need to wait for the next scheduled start date, which can lead to delays in enrolling in CR programs.

Providers also think that cultural restrictions can prevent some patients from enrolling in CR programs, particularly when there is discomfort or disallowance in mixed-gender sessions due to cultural or religious beliefs. Providers have mentioned that financial barriers are a concern for some patients and can affect their enrollment. Some patients may not have insurance coverage, or they may be unaware of what services their insurance will cover. Financial barriers can also include the costs associated with transportation and parking. In addition, in some CR centers, patients have the option to participate in CR programs virtually through videoconferencing and web-based meeting platforms such as Zoom (Zoom Video Communications). Providers mentioned a lack of technical knowledge and equipment requirements as factors contributing to nonenrollment in these virtual CR (VCR) programs. They noted that patients may encounter obstacles due to the need for reliable Wi-Fi, laptops, tablets, email addresses, and smartwatches to monitor vitals such as heart rate for VCR, which may not be accessible to all patients.

While providers have mentioned several factors for patients not enrolling in CR programs, they believe that some factors remain hidden and express uncertainty about fully understanding why some patients do not enroll in these programs. Providers have also highlighted that while they try to find these factors through patient data recorded in electronic medical records (EMRs), they are unable to fully explore and uncover the underlying reasons solely from EMRs due to missing or insufficient data. As one provider noted, “We try and capture what we can from [the EMR], so we can count visits and things like that within the EMR. It is definitely not perfect” [P7]. Providers expressed the need for a tracking system to understand potential hidden factors that contribute to patients not enrolling. As one provider mentioned, “[We] do need a little bit of like...something to keep track of, because there's quite a few patients that come through. So just to have...what date they start...it is nice to have an overview of the patient's journey” [P8].

### Patients Dropping Out of CR Programs

Dropping out of a CR program before completing it is also a barrier to progression in the CR patient journey, as it prevents patients from fully benefiting from the CR program (Table S3 in Multimedia Appendix 5). Providers observed that reproductive and hormonal conditions can deter female patients from completing or fully participating in CR programs. These conditions include concerns about the safety of exercise during pregnancy, managing pregnancy-related fatigue, menopause issues, and menstrual cycle discomfort, such as severe menstrual cramps. In addition, providers recognized that a lack of support is a factor for dropout, including insufficient family support to manage home obligations such as caregiving for children or older adult family members or a lack of peer support for mental and emotional well-being during the rehabilitation process.

Providers noted that low motivation, low self-efficacy, and a lack of belief in one’s ability to perform exercises or improve one’s lifestyle contribute to a lack of participation and eventual dropout from the program. As one provider mentioned, “They just don’t [participate] because they have no motivation to” [P8]. In addition, providers have observed that low accountability in some patients, requiring an external push and motivation from family or providers, can also lead to dropout. Although providers make efforts to encourage patients by emphasizing the benefits of continuing rehabilitation, some patients may still find it challenging to stay engaged with their rehabilitation, which can contribute to dropouts. As one provider mentioned, “We try to help them understand that if they [continue rehabilitation], their health can get better” [P9].

Patients may face several challenges in reaching CR centers, particularly when they lack access to a car or public transportation or when adverse weather conditions, such as extreme heat or cold, are present. Providers mentioned that these challenges in reaching CR centers can contribute to patient dropouts. In addition, the lack of multilingual support and translators in some CR programs can be challenging for patients who need services in other languages. Providers mentioned that these language barriers can also contribute to dropouts. Providers highlight that some patients may be frail due to medical conditions such as shortness of breath, arthritis, or chronic pain, which can prevent them from performing the required exercises, leading to frustration and dropout. Furthermore, in VCR, patients often need to interact with digital interventions for extended periods. Providers mentioned that this prolonged interaction can be one of the factors contributing to dropouts, particularly in VCR.

### Patients’ Lack of Adherence to Lifestyle Changes Post-CR Programs

Providers recommend that patients continue following the lifestyle changes they learned during the CR program even after finishing the CR program cycle to achieve better health-related quality of life. However, patients sometimes struggle to adhere to these recommendations, which becomes a barrier to progression in their journey (Table S4 in Multimedia Appendix 5). Providers noted that patients could experience persistent feelings of sadness, hopelessness, stress, and anxiety about daily life and responsibilities, which could lower their motivation and hinder their adherence to lifestyle changes. A lack of personal drive could also contribute to not adhering, as patients may struggle to set and achieve personal health goals independently. Providers noted that financial constraints may impact adherence to post-CR programs, with high costs for healthy food, gym memberships, medications, and inadequate insurance coverage preventing patients from fully following recommended lifestyle changes. Providers try to emphasize the importance of physical exercise in maintaining a healthy lifestyle, but only a small portion of patients follow this advice. As one provider noted, “The reality is that a small percentage of patients, once they graduate rehab, remain as physically active as they were during rehab” [P3]. In addition, providers mentioned that some patients face challenges in performing physical exercises due to factors such as lack of space at home, exercise-induced pain or discomfort, and a lack of resources to fully understand and perform exercises without pain or discomfort. These challenges lead to frustration and doubts about their ability to perform exercises, as mentioned by a provider: “I do get a few [patients] that tell me they're just not interested because exercise is just not for them” [P9]. Providers think that a lack of monitoring and follow-up clinical appointments could also contribute to nonadherence, with insufficient regular feedback on health progress and areas for improvement leading to decreased adherence to lifestyle changes.

## Discussion

### Technological Solutions

In this study, we explored the barriers to progression in the CR patient journey from the health care providers’ perspectives. We identified four barriers that hinder progression in the CR patient journey, such as (1) patients not being referred to CR programs, (2) patients not enrolling in CR programs, (3) patients dropping out of CR programs, and (4) patients’ lack of adherence to lifestyle changes post-CR programs. Based on our findings, we recommend 4 potential technological solutions to overcome these barriers: monitoring patients’ progress throughout the CR journey using interactive data visualization systems, monitoring physical exercises through automated video analysis–based feedback systems, matching the patients to the right programs using machine learning (ML)–driven predictive analysis, and supporting patients with self-management using targeted natural language processing (NLP) and large language model (LLM) tools. These potential solutions could provide the foundation for future research and guide the development of effective technologies to overcome barriers to progression in the CR patient journey.

#### Monitoring Patients’ Progress Throughout the CR Journey Using Interactive Data Visualization Systems

Health care providers have noticed that some patients in CR programs do not show adequate improvement in their progress, which could be due to the loss of motivation over time. In addition, they have mentioned that there may be other hidden reasons within the CR programs that are impacting patients’ progress, making it difficult for patients to take necessary actions to improve progression. Effective monitoring of the patient’s progress can help patients stay motivated [32-34] and help providers identify any hidden reasons affecting patients’ progress [35,36]. Research has shown that interactive data visualization systems are effective in monitoring patients’ progress for both patients [37,38] and providers [39,40]. Based on the success of these systems, we propose the development of interactive data visualization tools for monitoring patients’ progress in CR. Patients and providers have different goals and require varying levels of detail in monitoring patients’ progress [41]. Therefore, visualization systems should be developed targeting the unique needs of patients and providers with a human-centered approach [42] while involving them in the process of design and development.

For patients, visualization systems can enable them to monitor their progress throughout the CR journey by using EMR and personally collected health data in CR, such as vital signs, exercise performance, attendance, and assessment results. The system can include gamified visualizations [43] and real-life object representations [44] to make it engaging and easier for patients to understand their progress, rather than using complex charts. For example, a patient who visually sees an improvement in heart rate rhythm and blood pressure after regular exercise may feel more encouraged to actively participate throughout the CR journey.

For providers, visualization systems can assist them in monitoring the progress of patients in CR journey and help them identify any underlying reasons that affect patient progression by using aggregated EMR data of patients in the CR center. Providers can use the visualization system to filter and drill down into specific patients or groups based on demographic characteristics, particular cohorts, or specific CR sessions to analyze patients’ attendance patterns and improvements or declines in progress [45]. For instance, providers might discover a trend where patients older than 65 years show better progress in heart rate rhythm when exercise sessions are coupled with meditation than when they are offered solely.

#### Monitoring Physical Exercises Using Automated Video Analysis–Based Feedback Systems

Providers noticed that many patients in CR struggle to perform physical exercises correctly, particularly during VCR and after completing CR programs, where direct supervision from health care providers is not available. Without this supervision and real-time feedback, patients often find it challenging to understand proper exercise techniques, leading to uncertainty about their form and an increased risk of adopting incorrect postures or unsafe practices, which could result in injuries. Recent studies show promising results for monitoring physical exercises with artificial intelligence–powered feedback systems that analyze posture [46,47] and provide real-time exercise feedback through video analysis [48-50]. Building on the success of previous artificial intelligence–powered feedback systems, we suggest developing automated video analysis–based physical exercise feedback systems to guide and monitor patients’ exercise techniques and provide immediate corrective feedback.

The system can leverage pose estimation algorithms such as DensePose [51], YOLO-Pose [52], and OpenPose [53] to identify patient posture by detecting joints in real-time video. These algorithms can be trained on a dataset including correct and incorrect postures, as demonstrated and annotated by health care providers [54]. The system can then incorporate mathematical algorithms that calculate the angles between various body joints to classify whether a posture is correct or incorrect [47]. Patients can perform physical exercises while the system captures video through their web cameras. The system would analyze the video feed in real time, classifying the exercise form as correct or incorrect. If an incorrect form is detected, the system will identify the specific area of the body that is not aligned properly and provide immediate feedback. This feedback could be delivered through text and image overlays, offering suggestions such as “You are doing a great job, but bend more,” alongside visual corrections that highlight the areas needing adjustment. In addition, real-time voice prompts could be integrated to help patients quickly understand and adjust their form during the exercise. For example, if a patient is performing a lunge and their front knee extends too far forward over their toes, the system might display a red overlay on the knee area with a voice prompt saying, “Step back slightly to keep your knee aligned with your ankle.”

#### Matching the Patients to the Right Programs Using ML-Driven Predictive Analysis

Health care providers mentioned that patients often drop out early from CR programs, which could be because they are not matched to a program that meets their needs during referral [55,56]. Providers face challenges in identifying which patients are at risk of dropping out and selecting the appropriate program that aligns with those needs, as this process is complex and time-consuming. Previous research has demonstrated the efficacy of predictive algorithms in successfully predicting dropout rates and selecting appropriate programs. For instance, predictive algorithms have been used to predict dropout rates in health care programs [57,58], such as cognitive behavioral therapy for insomnia [59], chronic disease management programs [60], rehabilitation programs [61-63], and for selecting appropriate treatment plans [64,65]. Building on previous research, we suggest that ML-driven predictive analysis algorithms can be developed to proactively identify patients at heightened risk of dropping out from CR programs based on their characteristics and historical program outcomes and suggest the most suitable CR programs for each patient.

These algorithms can be developed using various ML models. For example, logistic regression [66] or naive Bayes models [67] can assess how specific patient factors influence the likelihood of dropout. Random forests [68], or eXtreme Gradient Boosting models [69], can be used to identify which factors (or combinations of factors) are most predictive of dropouts. Gradient boosting machines [70] or support vector machines [71] can then predict dropout risk and recommend the most suitable CR programs based on the identified factors. These algorithms can be trained using historical patient data such as age, gender, location, marital status, education level, socioeconomic status, comorbidities, and previous CR program and health outcomes (eg, completion, dropout, improvement in cardiac health, readmission, or relapse). Once trained, the algorithm can be used to match new patients to the right CR program at the point of referral. By comparing the characteristics of new patients with those of previous patients who dropped out, the algorithms can pinpoint factors associated with dropout risks, such as irregular attendance or poor performance in rehabilitation sessions. The algorithm can generate risk scores that indicate the new patients’ likelihood of dropout. It can then recommend the CR program that has historically yielded the best outcomes for patients with similar profiles. For instance, a 65-year-old patient with a history of diabetes and hypertension and a 90% dropout risk due to long travel distance might be matched with a program offering fewer inpatient visits or a virtual option, emphasizing dietary management and low-impact exercises based on the success of similar profiles in historical data.

#### Supporting Patients With Self-Management Using Targeted NLP and LLM Tools

Providers observed that many patients in CR face difficulties in self-management. This includes difficulties in independently setting their health goals and finding reliable answers to their health-related questions outside of their scheduled appointments with providers. To help patients set their health goals on their own, NLP-based automated goal-setting tools have been shown to be effective in various medical conditions such as hypertension [72], HIV [73], stroke [74], and aphasia [75]. Similarly, to help patients find answers to their health-related questions, LLM-based chatbot solutions have been shown to be successful, particularly, for patients managing chronic diseases [76], inflammatory bowel diseases [77], and breast cancer [78]. Building on the effectiveness of these tools in various medical conditions, we suggest developing targeted NLP- and LLM-based tools specifically for patients in CR. NLP-based tools, such as mobile apps or web interfaces, can assist patients in setting personal health goals in CR. LLM-integrated chatbots can help patients find answers to their questions and enhance their understanding throughout their CR journey.

For goal setting, NLP-based tools can allow patients to input detailed information about their health conditions, preferences, goals, and obstacles in their own words. Using NLP techniques, the tool can use intent recognition [79] to identify patient objectives, entity extraction [80,81] to categorize health conditions and challenges, and sentiment analysis [82,83] to assess emotional states and motivation levels. Based on this analysis, the technology can generate personalized goals using the SMART (Specific, Measurable, Achievable, Relevant, Time-bound) framework [84]. Patients can also review, refine, prioritize, and track their goals over time. For example, if a patient wants to “exercise more” but struggles with “low energy levels,” the technology could suggest a goal based on the SMART framework, such as “Walk for 20 minutes, three times a week, and gradually increase the duration as energy improves.”

To provide answers to patients’ questions, targeted LLM-based chatbots can be trained on CR-specific educational resources, a database of frequently asked questions by patients in CR, and anonymized patient-provider conversations specific to CR. The chatbot should be capable of providing CR education, detailed explanations to patients’ questions; offering empathetic responses and coping strategies for managing stress, anxiety, and other emotional challenges; and assisting patients in finding support groups and educational materials tailored to their needs. If patients have questions anytime throughout the CR patient journey, they can access the chatbot. For instance, if a patient asks how to safely continue their exercise routine at home while experiencing menstrual cramps, the chatbot could recommend gentle stretching exercises or advise reducing workout intensity while encouraging confirmation of this guidance with their provider at their next appointment [85].

### Limitations

While our study aimed to capture the perspectives of health care providers from various specialties involved in CR care, the use of snowball sampling may have introduced selection bias, as participants were primarily recruited based on a research collaboration network. Although we reached thematic saturation after 7 interviews, indicating common barriers across providers from different specialties, the small sample size of 10 participants may not fully reflect the diversity of perspectives, particularly as we did not include providers from other countries. In addition, despite following rigorous methodological standards, the qualitative analysis remains interpretative by nature and may be subject to researcher bias [84]. As with any qualitative study, the rigor of our findings should be judged by their resonance and plausibility rather than their generalizability. Furthermore, our findings may not be transferable to other CR centers that differ in resources, cultures, or geographical settings [85].

### Conclusions

The objective of this study was to explore the barriers to progression in the CR patient journey from the perspectives of health care providers involved in CR care. Our findings show that patients not being referred to CR programs, patients not enrolling in CR programs, patients dropping out of CR programs, and patients’ lack of adherence to lifestyle changes post-CR programs are the 4 barriers to progression as perceived by providers. We also proposed 4 potential technological solutions to overcome the barriers identified through our study. Future work should focus on designing, developing, and evaluating the technological solutions proposed in this study to overcome the barriers to progression in the CR patient journey.

## Appendix

Multimedia Appendix 1 COREQ (COnsolidated criteria for REporting Qualitative research) checklist.

Multimedia Appendix 2 Semistructured interview script and questions.

Multimedia Appendix 3 Representative quotes on barriers to progression in the cardiac rehabilitation patient journey.

Multimedia Appendix 4 Saturation table.

Multimedia Appendix 5 Codebook.

## Abbreviations

COREQ: COnsolidated criteria for REporting Qualitative research
CR: cardiac rehabilitation
EMR: electronic medical record
LLM: large language model
ML: machine learning
NLP: natural language processing
SMART: Specific, Measurable, Achievable, Relevant, Time-bound
VCR: virtual cardiac rehabilitation
