# Supplementary material for: Exploring Barriers to Patients’ Progression in the Cardiac Rehabilitation Journey From Health Care Providers’ Perspectives: Qualitative Study
Source: Interact J Med Res. 2025 Feb 21;14:e66164. doi: 10.2196/66164 (PMC11890148; doi:10.2196/66164)
Supplement: Multimedia Appendix 1 [file ijmr_v14i1e66164_app1.pdf]

Multimedia Appendix 1

COREQ (COnsolidated criteria for REporting Qualitative research) Checklist

Topic	Item No.	Guide Questions/Description	Reported on Page No.
**Domain 1: Research team and reflexivity**			
Personal characteristics			
Interviewer/facilitator	1	Which author/s conducted the interview or focus group?	2
Credentials	2	What were the researcher's credentials? E.g. PhD, MD	2
Occupation	3	What was their occupation at the time of the study?	2
Gender	4	Was the researcher male or female?	2
Experience and training	5	What experience or training did the researcher have?	2
Relationship with participants			
Relationship established	6	Was a relationship established prior to study commencement?	2
Participant knowledge of the interviewer	7	What did the participants know about the researcher? e.g. personal goals, reasons for doing the research	2
Interviewer characteristics	8	What characteristics were reported about the interviewer/facilitator? e.g. Bias, assumptions, reasons and interests in the research topic	2
**Domain 2: Study design**			
Theoretical framework			
Methodological orientation and Theory	9	What methodological orientation was stated to underpin the study? e.g. grounded theory, discourse analysis, ethnography, phenomenology, content analysis	3
Participant selection			
Sampling	10	How were participants selected? e.g. purposive, convenience, consecutive, snowball	2
Method of approach	11	How were participants approached? e.g. face-to-face, telephone, mail, email	2
Sample size	12	How many participants were in the study?	3
Non-participation	13	How many people refused to participate or dropped out? Reasons?	2

Setting			
Setting of data collection	14	Where was the data collected? e.g. home, clinic, workplace	2
Presence of nonparticipants	15	Was anyone else present besides the participants and researchers?	2
Description of sample	16	What are the important characteristics of the sample? e.g. demographic data, date	4
Data collection			
Interview guide	17	Were questions, prompts, guides provided by the authors? Was it pilot tested?	2,3
Repeat interviews	18	Were repeat interviews carried out? If yes, how many?	2
Audio/visual recording	19	Did the research use audio or visual recording to collect the data?	2
Field notes	20	Were field notes made during and/or after the interview or focus group?	2
Duration	21	What was the duration of the interviews or focus group?	2
Data saturation	22	Was data saturation discussed?	3
Transcripts returned	23	Were transcripts returned to participants for comment and/or correction?	3
**Domain 3: analysis and findings**			
Data analysis			
Number of data coders	24	How many data coders coded the data?	3
Description of the coding tree	25	Did authors provide a description of the coding tree?	4-6
Derivation of themes	26	Were themes identified in advance or derived from the data?	3
Software	27	What software, if applicable, was used to manage the data?	3
Participant checking	28	Did participants provide feedback on the findings?	3
Reporting			
Quotations presented	29	Were participant quotations presented to illustrate the themes/findings? Was each quotation identified? e.g. participant number	4-6
Data and findings consistent	30	Was there consistency between the data presented and the findings?	3

Clarity of major themes	31	Were major themes clearly presented in the findings?	3-6
Clarity of minor themes	32	Is there a description of diverse cases or discussion of minor themes?	3-6
