# Supplementary material for: Exploring Barriers to Patients’ Progression in the Cardiac Rehabilitation Journey From Health Care Providers’ Perspectives: Qualitative Study
Source: Interact J Med Res. 2025 Feb 21;14:e66164. doi: 10.2196/66164 (PMC11890148; doi:10.2196/66164)
Supplement: Multimedia Appendix 2 [file ijmr_v14i1e66164_app2.pdf]

Multimedia Appendix 2

Semi-structured interview script and questions

Introduction:

Thank you for agreeing to participate in this interview. Your insights and experiences are crucial to our study. Before we begin, we would like to provide a brief overview of our study objectives. The aim of this study is to identify barriers to progression in the cardiac rehabilitation patient journey from the perspective of healthcare providers. We will start by collecting some basic demographic information, followed by a discussion of your insights and experiences.

As noted in the consent form, your information will be anonymized to protect your identity. We will be recording this interview for accuracy. You may choose to keep your camera on or off, but please be aware that the audio recording is mandatory for participation. If any questions are unclear, feel free to ask for clarification. We have a small team here (2-3 people), with one person conducting the interview while others take notes and ask follow-up questions.

With that said, let's begin. We appreciate your participation and look forward to your valuable insights. I will now start the recording.

Interview Questions

1. Demographic questions
   a. Age: How old are you?
   b. Gender: How do you identify your gender?
   c. Specialty: What is your specific specialty or area of expertise?
   d. Years of Experience: How many years have you worked in your specialty area?
   e. Location: Is your practice located in a rural or urban area?
2. What is your role in the cardiac rehabilitation program?
   a. Can you elaborate on your specific responsibilities and duties within the program?
3. Could you describe the stages of a patient's cardiac rehabilitation journey?
   a. Provide an overview of the typical steps a patient goes through from initial referral to program completion.
   b. How is the patient's journey tracked and monitored throughout the rehabilitation process?
   c. What electronic systems or software does your program use for managing patient information (e.g., Electronic Medical Records or similar systems)?
4. Can you explain the process for referring patients to cardiac rehabilitation programs?
   a. Discuss the criteria or considerations for determining the most appropriate program for each patient.
   b. What challenges or barriers do you encounter in the referral process for cardiac rehabilitation?
   c. Do these challenges affect patient enrollment or participation in cardiac rehabilitation? If yes, please explain how referral challenges might influence a patient’s ability to enroll in or participate in the program.
5. Can you describe the enrollment process for patients in cardiac rehabilitation programs?
   a. Detail the steps involved, from patient registration to program initiation.
   b. What are major challenges in the patient enrollment process?

    c. Do these enrollment challenges impact patient participation in cardiac rehabilitation? If yes, please explain how problems during enrollment may affect a patient's ongoing participation in the program.
6. What are the major challenges affecting patient participation in cardiac rehabilitation?
    a. How do you track patients who drop out of the cardiac rehabilitation program? Explain the methods or systems used to monitor and follow up with patients who discontinue the program.
7. What challenges do patients face after completing the cardiac rehabilitation program?
    a. What strategies or methods do you use to monitor patients after they complete the program?
8. Do perceived challenges or barriers to participation and completion change based on the type of cardiac rehabilitation program (in-person, hybrid, or virtual)?
9. Which patient demographics or characteristics are more likely to experience difficulties in participating in cardiac rehabilitation programs? Identify specific patient groups (e.g., age, socioeconomic status, comorbidities) that face more significant challenges.
10. What specific strategies have been implemented in your cardiac rehabilitation program to improve patient outcomes?
    a. What were the outcomes of these strategies in terms of increased participation or improved patient results? What lessons were learned from these implementations?
11. Do you have any final thoughts or recommendations for future strategies or improvements in cardiac rehabilitation programs?
