# Supplementary material for: Exploring Barriers to Patients’ Progression in the Cardiac Rehabilitation Journey From Health Care Providers’ Perspectives: Qualitative Study
Source: Interact J Med Res. 2025 Feb 21;14:e66164. doi: 10.2196/66164 (PMC11890148; doi:10.2196/66164)
Supplement: Multimedia Appendix 3 [file ijmr_v14i1e66164_app3.pdf]

Multimedia Appendix 3

Representative quotes on barriers to progression in the CR patient journey

Themes	Description	Representative Quotes
**1. Patients not being referred to CR programs**	**Codes in this category include factors that contribute to patients not being referred to CR programs**	"referral definitely a barrier and they [referring providers] just don't refer….I would say…the one thing that they could is refer a little bit more often or at least refer and let us see as the patient qualify or not (P9)"
1.1. Unintentional bias	Referral biases related to health conditions, geography, age, and gender.	"So there was a lot of inherent bias in terms of who is being referred to rehab (P3)"<br>"So they [referring providers] think all this patient is too frail or this patient is too old, or this one leaves far, or so they they have assumptions. So that's I think that's the key barrier in that [referral] process." (P1)
1.2. Limited time	High number of patients or heavy workload making referral time-consuming.	"For sure it's a massive job and it takes a lot of time, so there a team of humans [referring providers] to generate the referrals. So that is a challenge with our time is so limited (P7)"
1.3. Lack of knowledge	Lack of knowledge of the various programs available at the CR center.	"I think they [referring providers] don't have knowledge of what cardiac rehab is. So they don't really understand if their patients are, they will benefit from cardiac rehab." (P1)
1.4. Finding appropriate programs	Challenges in identifying the best CR program for each patient during referral without access to a wholesome list of CR programs	"Uh, currently, I would just say more or less the staff on the floors who refer don't understand which program to refer to or which is helpful (P8)"
**2. Patients not enrolling in CR programs**	**Codes in this category include factors that contribute to patients not enrolling in CR programs**	"I think it's about very few people that are referred may choose to enroll, very less people come to us (P5)."
2.1. Lack of awareness and knowledge	Patients being unaware of the importance and benefits of CR, patients hold misconceptions about CR, believing they can recover by themselves at home, belief that treatment alone is sufficient, and rehabilitation will not provide additional benefits.	"We do have a lot of, I would say, education barriers that people don't believe exercise is gonna help them or that they believe that they're too sick or they don't need it or I'm moving a lot, because a lot of people want the quick fix. They want the pill, they want the quick fix. (P5)"

2.2. Inconvenient waiting periods	Offering the program too soon after surgery when patients are not ready, prolonged waiting periods between referral and program start, programs offered in fixed schedules causing delays for patients who miss the enrollment window.	"Umm, I also think if it's not immediate referral they might not come back and they they will start to get better in two months, right? So it's like then you take two months to start the program and you tell them, oh, you're gonna start in two months. They're like, oh, maybe let me check. Another is, patients had to wait to join a certain batch , because they have to wait for the next batch to start(P1)"
2.3. Financial barriers	Lack of insurance coverage, patients' lack of knowledge about what their insurance will cover, costs associated with transportation and parking.	"it's more challenging if you're poor, getting into care, participating fully in care, getting diagnostics, getting procedures and then having good outcomes can be challenging for poor. (P4) "<br>"Some of them don't have vehicles, some of them can't even afford a bus pass to get to the hospital. (P2)"
2.4. Cultural restrictions	Discomfort or disallowance in participating in sessions with mixed genders due to cultural or religious beliefs.	"we were feeling that you know, there's certain cultures that women are not permitted to exercise with men in the same class. (P2)"
2.5. Lack of technical knowledge and equipment requirements	Need for laptops, iPads, or other medical devices such as vital monitors to participate in VCR, need for an email address and reliable WiFi are required, which may not be accessible for all patients.	"There's a huge challenge with technology in that patient population, for sure. But there's this barrier of the interface, and there's definitely not enough resource in the system to support that (P6)"<br>"not all of them had email addresses, not all of them had laptops. Right? a lot of them didn't have blood pressure machines, automated blood pressure machines at home, and they didn't have the equipment to be able to monitor (P2)"
2.6. Uncertainty about reasons for low enrollment	Lack of data on why certain patients do not participate.	"you don't know the reasons why they are not enrolling because we don't have data often mentioned (P7)"
**3. Patients dropping out of CR programs**	**Codes in this category include factors that contribute to patients dropping out from CR programs**	"They just drop out, there are lot of different reasons. we've had people that, you know, people gonna be able to do the program and then they get halfway through and it they don't.(P9)"
3.1. Reproductive and hormonal conditions	Concerns about the safety of exercise during pregnancy, managing pregnancy-related fatigue, menopause issues, and menstrual cycle discomfort, such	"So they [women] have …different risk factors, … they have multiple responsibilities, sometimes it's for other reasons, like there is the menopause and all those things that happened. So pregnancy related complications. So they miss classes more than men (P1) "

	as severe menstrual cramps.	
3.2. Lack of support	Lack of family support to take over obligations at home, such as caregiving for children or elderly family members, lack of peer support.	"Umm, I'm just basically thinking that it was that whole support from family or group feeling that was lacking and that's what keeps bringing them in, cause there's family and friendships that are formed with the other participants cause they've all gone through something similar, right? (P2) "
3.3. Low self-efficacy	Patients' low belief in their capacity to perform exercises or their ability to get a better lifestyle from the program	"Some patients don't believe they have the ability to improve… most of the patients who I get a few who just tell me they're just not interested because some people just don't wanna come and then I get all kind of different reasons (P6)"
3.4. Low accountability	Patients feeling unmotivated to adhere to the program without regular check-ins on their progress and failing to monitor their own progress can lead to non-completion.	"Dropout rate was high because there wasn't accountability by patients, right? (P2)"
3.5. Challenges in reaching CR centers	Issues organizing reliable transportation, long distances to CR centers, expensive and insufficient parking at CR centers, and adverse weather conditions, being too hot or too cold.	"the distance that they have to drive to come, like if you don't have a car, You have to rely on someone to come to the hospital and get to your appointment like we have a lot of those barriers for sure. there's also the weather, I would say, like during winter time, I do have more drop rate because if there's storm and stuff like that that people don't wanna come … I would see that people are dropping a lot. (P5)"
3.6. Cognitive fatigue	Cognitive fatigue due to extended interaction with screens in VCR programs.	"staring at a screen for long periods during virtual sessions, you know that can be exhausting for patients (P6)"
3.7. Language barriers	Lack of multilingual support, no translator in the on-site CR program, limited patient proficiency in the program's offered language.	"People with culturally and linguistically diverse background. So like if someone does not speak English, it's harder for them to come to understand. (P1)"
3.8. Frailty	Inability to perform exercises because of other medical conditions such as shortness of	"so lots of comorbidities, high burden of challenges just to get here and perform because they have dialysis and you have, you know arthritis, they maybe use a Walker, but they're they're the ones that don't perform exercise (P7)"

	breath, arthritis, or chronic pain.	
**4. Patients' lack of adherence to lifestyle changes post-CR programs**	**Codes in this category include factors that contribute to patients' lack of adherence to lifestyle changes post-CR programs**	"we really tell them to follow, but they just don't follow after you know after they graduate (P3)"
4.1. Lack of motivation	Persistent feelings of sadness, hopelessness, stress, depression and anxiety about daily life and responsibilities.	" there's so many fears and anxieties that they have that I think one of the barriers after program, And like I say, I do just get a few that tell me they're just not interested because exercise is just not for them (P9)"
4.2. Lack of personal drive	Absence of personal drive in setting and achieving personal health goals.	"they're just not interested and they want someone to help them always for all, you know for goals but we can't be always be there (P4) "
4.3. Financial constraints	Financial constraints that may prevent patients from affording healthy food options, gym memberships, medications and lack of insurance coverage.	"It's just the cost with everything, sometimes they can't afford for you know sometimes the food, or gym (P10)"
4.4. Lack of adherence to exercises	Exercise-induced pain or discomfort and insufficient resources or space for effective home-based exercise.	"And that's really where we try to push patients because we want them to maintain the behaviors and the reality is that a small percentage of patients, once they graduate rehab, remain as physically active as they were during rehab. (P3)"
4.5. Lack of monitoring and follow-ups	Lack of regular feedback on progress and areas for improvement from providers.	"So it's a lot more of like just kind of self monitoring after program and we kind of go based on what they should do at home but they need monitoring by us daily, that support and making sure that we're watching what they're doing at home (P9)"
