# Supplementary material for: Exploring Barriers to Patients’ Progression in the Cardiac Rehabilitation Journey From Health Care Providers’ Perspectives: Qualitative Study
Source: Interact J Med Res. 2025 Feb 21;14:e66164. doi: 10.2196/66164 (PMC11890148; doi:10.2196/66164)
Supplement: Multimedia Appendix 4 [file ijmr_v14i1e66164_app4.pdf]

Multimedia Appendix 4
Saturation table

Themes	Sub theme	P1	P2	P3	P4	P5	P6	P7	P8	P9	P10
1. Patients not being referred to CR programs	1.1. Unintentional bias	x		x			x		x		
	1.2. Limited time				x			x		x	
	1.3. Lack of knowledge	x				x		x	x		x
	1.4. Finding appropriate programs							x	x		
2. Patients not enrolling in CR programs	2.1. Lack of awareness and knowledge		x			x				x	x
	2.2. Inconvenient waiting periods	x			x		x				
	2.3. Financial barriers		x	x	x					x	
	2.4. Cultural restrictions		x								
	2.5. Lack of technical knowledge and equipment requirements		x				x	x			x
	2.6. Uncertainty about reasons for low enrollment							x			x
3. Patients dropping out of CR programs	3.1. Reproductive and hormonal conditions	x								x	
	3.2. Lack of support		x			x					
	3.3. Low self-efficacy				x		x				x
	3.4. Low accountability		x						x		
	3.5. Challenges in reaching CR centers	x		x	x	x					
	3.6. Cognitive fatigue		x				x				
	3.7. Language barriers	x								x	x
	3.8. Frailty							x			
4. Patients' lack of adherence to lifestyle changes post-CR programs	4.1. Lack of motivation				x				x	x	
	4.2. Lack of personal drive				x	x					
	4.3. Financial constraints	x								x	x
	4.4. Lack of adherence to exercises			x		x					
	4.5. Lack of monitoring and follow-ups			x					x		
