# Supplementary material for: Exploring Barriers to Patients’ Progression in the Cardiac Rehabilitation Journey From Health Care Providers’ Perspectives: Qualitative Study
Source: Interact J Med Res. 2025 Feb 21;14:e66164. doi: 10.2196/66164 (PMC11890148; doi:10.2196/66164)
Supplement: Multimedia Appendix 5 [file ijmr_v14i1e66164_app5.pdf]

Multimedia Appendix 5
Codebook

Table S1. Factors that contribute to patients not being referred to CR programs

Code	Description
**1. Patients not being referred to CR programs**	**Codes in this category include factors that contribute to patients not being referred to CR programs**
1.1. Unintentional bias	Referral biases related to health conditions, geography, age, and gender.
1.2. Limited time	High number of patients or heavy workload making referral time-consuming.
1.3. Lack of knowledge	Lack of knowledge of the various programs available at the CR center.
1.4. Finding appropriate programs	Challenges in identifying the best CR program for each patient during referral without access to a wholesome list of CR programs

Table S2. Factors that contribute to patients not enrolling in CR programs

Code	Description
**2. Patients not enrolling in CR programs**	**Codes in this category include factors that contribute to patients not enrolling in CR programs**
2.1. Lack of awareness and knowledge	Patients being unaware of the importance and benefits of CR, patients hold misconceptions about CR, believing they can recover by themselves at home, belief that treatment alone is sufficient, and rehabilitation will not provide additional benefits.
2.2. Inconvenient waiting periods	Offering the program too soon after surgery when patients are not ready, prolonged waiting periods between referral and program start, programs offered in fixed schedules causing delays for patients who miss the enrollment window.
2.3. Financial barriers	Lack of insurance coverage, patients' lack of knowledge about what their insurance will cover, costs associated with transportation and parking.
2.4. Cultural restrictions	Discomfort or disallowance in participating in sessions with mixed genders due to cultural or religious beliefs.
2.5. Lack of technical knowledge and equipment requirements	Need for laptops, iPads, or other medical devices such as vital monitors to participate in VCR, need for an email address and reliable WiFi are required, which may not be accessible for all patients.
2.6. Uncertainty about reasons for low enrollment	Lack of data on why certain patients do not participate.

Table S3. Factors that contribute to the patients dropping out of CR programs

Code	Description
**3. Patients dropping out of CR programs**	**Codes in this category include factors that contribute to patients dropping out from CR programs**
3.1. Reproductive and hormonal conditions	Concerns about the safety of exercise during pregnancy, managing pregnancy-related fatigue, menopause issues, and menstrual cycle discomfort, such as severe menstrual cramps.
3.2. Lack of support	Lack of family support to take over obligations at home, such as caregiving for children or elderly family members, lack of peer support.
3.3. Low self-efficacy	Patients' low belief in their capacity to perform exercises or their ability to get a better lifestyle from the program
3.4. Low accountability	Patients feeling unmotivated to adhere to the program without regular check-ins on their progress and failing to monitor their own progress can lead to non-completion.
3.5. Challenges in reaching CR centers	Issues organizing reliable transportation, long distances to CR centers, expensive and insufficient parking at CR centers, and adverse weather conditions, being too hot or too cold.
3.6. Cognitive fatigue	Cognitive fatigue due to extended interaction with screens in VCR programs.
3.7. Language barriers	Lack of multilingual support, no translator in the on-site CR program, limited patient proficiency in the program's offered language.
3.8. Frailty	Inability to perform exercises because of other medical conditions such as shortness of breath, arthritis, or chronic pain.

Table S4. Factors that contribute to patients' lack of adherence to lifestyle changes post-CR programs

Code	Description
**4. Patients' lack of adherence to lifestyle changes post-CR programs**	**Codes in this category include factors that contribute to patients' lack of adherence to lifestyle changes post-CR programs**
4.1. Lack of motivation	Persistent feelings of sadness, hopelessness, stress, depression and anxiety about daily life and responsibilities.
4.2. Lack of personal drive	Absence of personal drive in setting and achieving personal health goals.
4.3. Financial constraints	Financial constraints that may prevent patients from affording healthy food options, gym memberships, medications and lack of insurance coverage.
4.4. Lack of adherence to exercises	Exercise-induced pain or discomfort and insufficient resources or space for effective home-based exercise.
4.5. Lack of monitoring and follow-ups	Lack of regular feedback on progress and areas for improvement from providers.
